# Psychometric Evaluation of the Brazilian Version of the Diabetic Foot Self-Care Questionnaire of the University of Malaga-Spain (DFSQ-UMA-Br)

**DOI:** 10.3390/nursrep16050160

**Published:** 2026-05-09

**Authors:** Amelina de Brito Belchior, Victória Maria Silva Leitão, Thiago Martins de Sousa, Lourival Veras de Oliveira, Pablo Casimiro Belchior Rodrigues, Florencia Gamileira Nascimento, Sherida Karanini Paz de Oliveira

**Affiliations:** 1 Graduate Program in Clinical Care in Nursing and Health, Universidade Estadual do Ceará, Av. Dr. Silas Munguba, 1700—Campus do Itaperi, Fortaleza 60714-903, CE, Brazil; amelinabelchior@hotmail.com (A.d.B.B.); lourival.oliveira@aluno.uece.br (L.V.d.O.); florencianascimento@gmail.com (F.G.N.); sherida.oliveira@uece.br (S.K.P.d.O.); 2 Department of Nursing, Universidade Estadual do Ceará, Av. Dr. Silas Munguba, 1700—Campus do Itaperi, Fortaleza 60714-903, CE, Brazil; victoria.leitao@aluno.uece.br; 3 Department of Medicine, Universidade Regional do Cariri (URCA), Campus Madre Feitosa, Rua Coronel Antônio Luiz, Pimenta, Crato 63105-000, CE, Brazil; pablo.belchior@urca.br

**Keywords:** validation studies, surveys and questionnaires, psychometrics, diabetes mellitus, diabetic foot, reliability, self-care, nursing

## Abstract

**Objective**: To test the factorial structure and reliability of the Brazilian version of the Diabetic Foot Self-Care Questionnaire of the University of Malaga-Spain (DFSQ-UMA-Br). **Method**: Cross-sectional study for psychometric evaluation of the DFSQ-UMA-Br conducted with 269 people with diabetes who responded to the items of the collection instrument composed of sociodemographic and clinical data and the tested instrument, from February 2024 to February 2025. The internal structure was evaluated using Exploratory and Confirmatory Factor Analysis. Reliability was assessed using Cronbach’s alpha and McDonald’s omega. **Results**: The factor analysis for the DFSQ-UMA-Br data matrix was adequate (KMO = 0.79 [95% CI] = 0.71–0.82]); Bartlett’s sphericity test = *p* < 0.001; degree of freedom of 66), indicating that the variables are correlated. Psychometric sensitivity with adequate values of asymmetry (sk = 0.199–3.655) and kurtosis (ku = −0.226–+3.764) proving the normal distribution of data. **Conclusions**: The DFSQ-UMA-Br is a valid and reliable instrument capable of assessing foot self-care in people with diabetes in the Brazilian population and can be used by nurses and other professionals to promote health in the context of clinical care and research.

## 1. Introduction

Diabetes-related foot disease is among the most serious complications of diabetes, compromising the quality of life for those affected. It is a complex but preventable problem that causes intense suffering and entails high financial costs for patients, their families, healthcare professionals, healthcare systems, and society [1].

It is a microvascular complication characterized by the presence of infection, ulceration, and/or destruction of deep tissues, which substantially increases morbidity and mortality [2]. In addition, this condition encompasses one or more of the following symptoms present in the feet of people with diabetes mellitus (DM), diagnosed at any time in their lives: peripheral neuropathy, peripheral arterial disease, infection, ulcer(s), neuro-osteoarthropathy, gangrene, or amputation [3].

Infection of the feet is common and potentially devastating, developing in more than half of all foot ulcers and being the factor most frequently associated with lower-limb amputation [3].

It is estimated that 131 million people worldwide have lower-limb complications associated with diabetes, corresponding to 1.8% of the global population [4,5]. This growing number, combined with the difficulties of clinical management, limitations in health services, and social inequalities, results in unacceptable amputation rates [6].

Among these complications, 85% of lower-limb amputations performed in people with diabetes are preceded by a foot ulcer. The 5-year mortality rate is 2.5 times higher than the risk for a person with diabetes who does not have a foot ulcer [3]. In these individuals, the incidence of a new lower-limb amputation is higher among those who have undergone previous amputations [7].

However, foot ulcers in people with diabetes can be prevented with proper monitoring and evaluation. Therefore, every effort should be made to prevent them, as they are precursors to amputation and mortality [1].

In this sense, to reduce the impact of diabetes-related foot disease, strategies are needed that include prevention, patient and staff education, standardized assessment and classification, multidisciplinary treatment, and rigorous monitoring. In addition, five elements as part of integrated care for people with diabetes mellitus (DM) should be addressed, namely: 1—Identifying individuals with at-risk feet, 2—Inspecting and examining the feet of individuals at risk of foot ulceration, 3—Providing structured education for patients, their families, and healthcare professionals, 4—Encouraging the routine use of appropriate footwear, 5—Treating risk factors for foot ulceration. In addition, regular foot assessments should be performed routinely and systematically by healthcare staff, who need to be trained [1]. Early recognition and treatment of people with feet at risk for developing lesions and amputations can delay or prevent these complications, which are common and represent the main causes of morbidity and mortality in people with DM [7,8]. Thus, self-care can be considered a strategy to prevent foot complications in people with diabetes.

According to Orem’s Self-Care Theory, self-care is defined as the practice of activities that individuals initiate and perform on their own behalf to maintain life, health, and well-being. When individuals become unable to meet their own needs, healthcare professionals intervene to support and empower them to perform self-care [9].

Given the high epidemiological rates, the magnitude of diabetes-related foot disease for individuals, society, and the healthcare system, and the importance of self-care in preventing this complication, it is important to monitor the effectiveness of self-care interventions using validated measurement instruments that provide reliable, useful data.

In a scoping review conducted by Belchior et al. (2023) [10], eight instruments specific to foot self-care were identified, including one Brazilian instrument whose construct focused on adherence to self-care. Therefore, there are no Brazilian instruments that assess foot self-care behavior in people with diabetes. Furthermore, no studies using Patient-Reported Outcomes (PROs) were identified in Brazil [10].

The lack of instruments to assess foot self-care behavior in people with diabetes reveals a gap that needs to be addressed in the literature.

In Spain, the Diabetic Foot Self-Care Questionnaire of the University of Malaga-Spain (DFSQ-UMA) was originally developed and validated in Spanish. This questionnaire for assessing foot self-care in people with diabetes is designed to detect self-care deficits, which can be completed independently by the patient [11]. To date, cross-cultural adaptation has also been carried out, with evidence showing good psychometric properties of the DFSQ-UMA for French (DFSQ-UMA-FR) [12], Italian (SDFQ-IT) [13], Persian [14], Arabic (DFSQ-AR) [15], English (DFSQ-UMA-En) [16], and Turkish (DFSQ Turkish version) [17].

The Brazilian version of the Diabetic Foot Self-Care Questionnaire of the University of Malaga, Spain (DFSQ-UMA-Br) has demonstrated validity for assessing foot self-care behavior in people with diabetes, as demonstrated by the Patient-Reported Outcomes—PRO. The DFSQ-UMA-Br obtained a content validity index of 0.99 with a binomial test confirming that there was no significant disagreement among experts [18]. To date, no studies have been found that analyzed the psychometric properties of the DFSQ-UMA-Br [10].

However, for an instrument to be applied in clinical practice and research, it must be evaluated for its psychometric properties. If the validity and reliability of the measurement tools are not established, the results will be invalid and will not advance the development of nursing theory and practice [19].

Thus, the use of a valid and reliable instrument to measure self-care in this population will enable quality health care and the development of a care plan tailored to specific individual needs, in addition to motivating and guiding self-care for people with DM.

Given the above, this study aimed to test the factorial structure and reliability of the Brazilian version of the Diabetic Foot Self-Care Questionnaire (DFSQ-UMA-Br) developed by the University of Malaga, Spain.

## 2. Materials and Methods

### 2.1. Study Design

This is a cross-sectional study conducted in accordance with the Standards for Educational and Psychological Testing [20], and the Strengthening the Reporting of Observational Studies in Epidemiology (STROBE) [21].

### 2.2. Location and Data Collection Period

The study was conducted in three Specialized Centers for Diabetes and Hypertension Care (CEADH), secondary health care services in Fortaleza, Ceará, Brazil, from February 2024 to February 2025.

### 2.3. Population, Selection Criteria, and Sample Definition

Participants were selected using a consecutive sampling strategy, in which all eligible individuals attending the participating services during the data collection period were invited to participate. This approach was adopted to reduce selection bias and to ensure that the sample reflected the clinical and sociodemographic diversity of individuals with diabetes treated in secondary care. Eligibility was determined based on predefined inclusion and exclusion criteria, applied systematically by the research team at the time of recruitment.

Considering the general item pool and with the intention of preserving heterogeneity and obtaining respondents who would cover the entire construct, the sample was defined as a minimum of 15 to 20 participants per item of the DFSQ-UMA-Br instrument [22]. Thus, a minimum of 200 participants was calculated. The sample size for psychometric studies is estimated based on the number of items, which indicate ratios of 10:1 or higher [22]. This explains why the number of participants was considered adequate based on recommendations in the literature.

As selection criteria, individuals aged 18 years or older, with any type of diabetes, and receiving regular follow-up care at the CEADH were included. The exclusion criteria were individuals without access to a telephone for contact and those with bilateral foot amputation.

In addition to the rule of 15 to 20 participants per item, the sample size was also considered adequate based on psychometric recommendations for factor analysis, which indicate that samples above 200 participants provide stable factor solutions, particularly for instruments with moderate communalities and well-defined factor structures. Furthermore, considering the complexity of the model and the estimation methods employed (e.g., polychoric correlations and robust extraction techniques), this sample size ensures sufficient statistical power to detect factor loadings of practical relevance and to produce reliable goodness-of-fit indices. Thus, the final sample size was deemed adequate for both exploratory and confirmatory factor analyses.

### 2.4. Data Collection Instruments and Study Variables

The data collection instruments consisted of two parts: (1) a structured questionnaire to collect sociodemographic and clinical data; (2) the DFSQ-UMA-Br.

To collect sociodemographic and clinical data, a questionnaire was administered with the following variables: age, gender, marital status, religion, skin color (self-reported), education level, occupation, number of children, number of people living in the household, individual and family income, type and duration of diabetes, family history of DM, and type of family DM. Variables related to treatment and self-care were also included, such as type of treatment (oral antidiabetics and/or insulin), specific types of medications used, frequency of insulin administration, non-pharmacological treatment, presence of assistance in diabetes treatment, and who provides this support. Information was also collected on habits and self-care, including physical activity, specific diet for diabetes, smoking, frequency of capillary blood glucose testing, date and value of the last capillary blood glucose test, weight, height, and value of the last glycated hemoglobin test. Regarding clinical conditions, acute and chronic complications, presence of comorbidities, history of diabetic foot ulcers or amputations, specialists with whom clinical follow-up is performed, vision impairment, and receipt of assistance with foot care, the latter specifying who provides it.

The DFSQ-UMA-Br is a tool for assessing foot self-care with value in clinical practice. It is self-administered with 16 items distributed across three important domains of foot self-care: 1—Self-care; 2—Self-management and self-examination; 3—Footwear (shoes and socks).

The questions are arranged by domain or subscale, with five response options ranging from A to E. Option “A” reflects the best foot self-care behavior and is equivalent to five (5) points; option “E” corresponds to the worst self-care and is equivalent to one (1) point [23].

Although the original DFSQ-UMA-Br is structured into three theoretical domains (self-care, self-management/self-examination, and footwear), the present study also explored the internal structure empirically to verify whether this multidimensional configuration would be supported in the analyzed sample. Therefore, subsequent analyses considered the possibility of alternative factorial solutions, including unidimensional models, based on empirical fit indicators and parsimony criteria.

It is noteworthy that the author of the original instrument authorized its translation, adaptation, validation, and use in Brazil.

### 2.5. Data Collection

During data collection, patients were approached and informed about the study objectives and their participation. After accepting, they signed the Free and Informed Consent Form (FICF) and were interviewed using a semi-structured instrument with sociodemographic and clinical data, after which they were given the DFSQ-UMA-Br.

Data collection was performed by doctoral students in Clinical Nursing and Health Care and undergraduate nursing students at the State University of Ceará (UECE). The first application of the instrument (test) was performed on site, before or after the consultation, at the time indicated by the patient.

The second administration was conducted by telephone, between two and six weeks after the first test administration. This interval was chosen to balance potential methodological biases. Very short intervals may favor memory effects, leading to an overestimation of reliability, whereas longer intervals increase the likelihood of real changes in the construct being measured. Therefore, the adopted time frame is consistent with methodological recommendations in the literature and is considered appropriate for assessing the temporal stability of measures in clinical and behavioral contexts [24,25].

### 2.6. Data Processing and Analysis

All statistical calculations followed established psychometric procedures. Factor extraction was based on polychoric correlation matrices due to the ordinal nature of the data. Reliability indices (Cronbach’s alpha, McDonald’s omega, and composite reliability) were calculated using standardized formulas based on item variances and factor loadings. Goodness-of-fit indices were computed according to conventional structural equation modeling equations, and their interpretation followed widely accepted thresholds reported in the literature.

The psychometric properties of the DFSQ-UMA-Br were evaluated by examining its internal structure, its relationships with external measures, and the item response process.

Initially, we sought to test the pre-specified theoretical model using Exploratory Factor Analysis (EFA), a multivariate technique used to identify latent structures that explain the relationships among observed variables in a data set [21]. In this analysis, the suitability of the data for factor analysis was evaluated using the Kaiser–Meyer–Olkin value (KMO > 0.60)—to indicate suitability—and Bartlett’s sphericity test. If the calculated probability of error (*p*-value) of the sphericity test is below 0.05, it is concluded that the correlation or covariance matrix is not a unitary matrix; therefore, factor analysis can be applied [26].

Parallel Analysis was used to evaluate the instrument’s dimensionality using the Parallel Analysis Optimal Implementation technique [26], with robustness ensured by applying the bootstrap method and extrapolating the sample to 5000 cases to estimate confidence intervals for the evaluated parameters [27].

The factors were extracted using the Robust Unweighted Least Squares (ULS) method, with polychoric correlation and Robust Promin rotation [28]. To further analyze the number of factors in the instrument, unidimensionality/multidimensionality indicators were used: Unidimensional Congruence (UniCo > 0.95), Explained Common Variance (ECV > 0.85), and Mean of Item Residual Absolute Loadings (MIREAL < 0.30) [29].

The decision to retain or exclude items from the model considered the following criteria: convergence of the polychoric matrix; percentage of covariance destroyed per item (PCDi); correlation greater than 0.2 with at least two other items (those that did not meet this criterion were eliminated); absolute values of kurtosis (ku) and skewness (sk) less than 3 and 7, respectively, to ensure the assumption of normal data distribution [30]; communalities (h^2^ > 0.40); and factor loadings > 0.30. Furthermore, items with Heywood cases (factor loadings ≥ 1.00) and double saturation were excluded [22].

Although EFA and CFA are ideally conducted in independent samples, both techniques were applied to the same dataset due to sample size constraints. This approach is acceptable in initial validation studies and allows for preliminary testing of the factorial structure, provided that results are interpreted with caution and supported by multiple fit and reliability indicators.

Next, factor validity was tested using Confirmatory Factor Analysis (CFA), with the following indices to assess the quality of the model fit: Tucker–Lewis Index (TLI > 0.90); Comparative Fit Index (CFI > 0.94); Goodness of Fit Index (GFI > 0.95); Standardized root mean square residuals (SRMR) and Root Mean Square Error of Approximation (RMSEA < 0.07) [31].

The quality and effectiveness of the estimates were assessed in terms of accuracy (Overall Reliability of fully Informative prior Oblique N-EAP scores—ORION > 0.70); representativeness of the latent trait and effectiveness of the factor estimate (Factor Determinacy Index—FDI > 0.80 [29]; sensitivity (Sensitivity Ratio—SR > 2.0); expected percentage of the factor (Expected Percentage of true Differences—EPTD > 90%) and replicability (Generalized G-H Index > 0.80) [29].

These indices were selected because they provide complementary information about the quality of factor score estimates, replicability, and construct representation, going beyond traditional fit indices. Their use is recommended in contemporary psychometric studies to ensure more robust evaluation of latent structures.

The reliability of the factors was verified using Cronbach’s alpha coefficient, using the Psych statistical package of R, and using McDonald’s omega (ω) reliability and composite reliability (CR), calculated using the Composite Reliability Calculator, based on standardized factor loadings and error variations (https://thestatisticalmind.com/comp-reliability/, accessed on 26 April 2026). Values ≥ 0.70 are considered satisfactory in exploratory studies [32].

The questionnaire was tested to assess the adequacy of the measurement process, to investigate whether similar results are obtained when the instrument is applied under the same methodological conditions at different times [33].

The test–retest stability of the items was assessed using weighted Kappa, with 95% confidence intervals estimated. To this end, the cut-off points for classifying the level of stability of the responses were: weak (0 to 0.20); mild (0.21 to 0.40); reasonable (0.41 to 0.60); good (0.61 to 0.80); very good (0.81 to 0.92); excellent (0.93 to 1.00) [34].

During data processing and analysis, statistical tests were used in an integrated manner to investigate the psychometric properties of the DFSQ-UMA-Br items. According to [35], this approach not only allows analysis of the construct structure but also enables empirical validation of its consistency.

Statistical analyses were performed using the statistical programs Factor (version 12.03.02) and R (version 3.6.2).

### 2.7. Ethical Considerations

The study complied with the principles and legal requirements of Resolution No. 466/2012 of the National Health Council and was approved by the Research Ethics Committee (CEP) of the State University of Ceará under opinion number: 6,596,114 and CAAE: 72787023.1.0000.5534. All participants signed the Informed Consent Form in duplicate.

## 3. Results

The study sample consisted of 269 participants, aged between 20 and 92 years (median = 61; p25–p75 = 54–68; *p* = 0.01). There was a predominance of females (171; 63.6%), married or in a stable relationship (141; 52.4%), and self-declared brown skin (186; 69.1%). Regarding education, 34.9% (94) had an incomplete elementary school education, and 22.3% (60) had completed high school. More than half of the participants were retired (141; 52.4%), with a median of two children (p25–p75 = 2–4) and three people residing in the same household (p25–p75 = 2–4). Individual income was mostly concentrated at up to one minimum wage (119; 44.2%), with no information available in 49.8% (134) of cases.

Regarding the type of diabetes, T2DM was the most prevalent (249; 92.6%), with diagnosis time ranging from 0 to 50 years (median = 10; p25–p75 = 5–19). Most reported a family history of diabetes (224; 83.3%), mainly type 2 (204; 75.8%). Regarding treatment, 88.5% (238) used oral antidiabetic drugs, mainly metformin (214; 79.6%), and 58% (156) used insulin, with a median of three daily applications (p25–p75 = 2–4). Non-pharmacological treatment was rarely mentioned (1; 0.4%), and 46.1% (124) reported receiving help with treatment, mainly from children (63; 23.4%) and spouses (54; 20.1%). Complications were reported by 61.3% (165) of participants, with 18.2% (49) being acute and 59.5% (160) chronic; 13% (35) had associated comorbidities. Foot ulcers were observed in 13.4% (36) of cases, and 14.5% (39) had a history of this condition. In addition, 72.1% (194) reported receiving help with foot care, mainly from close family members, such as children (32; 11.9%) and spouses (27; 10%).

The factor analysis for the DFSQ-UMA-Br data matrix was adequate (KMO = 0.79 [95% CI] = 0.71–0.82]); Bartlett’s sphericity test = *p* < 0.001; degree of freedom of 66), indicating that the variables are correlated. Psychometric sensitivity with adequate values of asymmetry (sk = 0.199–3.655) and kurtosis (ku = −0.226–+ 3.764) (Table 1), confirming the normal distribution of the data.

**Figure 1:**
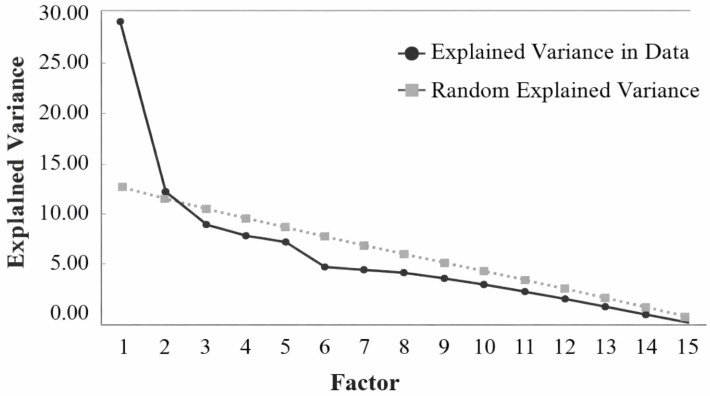
Explained Variance.

The sedimentation diagram indicated a representative factor for the item, responsible for the explained variance of the data (Figure 1).

Table 2 corroborates the sedimentation diagram, confirming the unidimensionality of the instrument, using the UniCo (0.994; 95% CI = 0.734–0.999), ECV (0.857; 95% CI = 0.783–0.992), and MIREAL (0.200; 95% CI = 0.183–0.206).

In analyzing load saturation, it was observed that the commonalities were adequate. There was no violation of the factor load limit (Heywood cases: −1 + 1), and all were saturated in a single dimension. Collinearity was observed in item 08 (>0.85) (Table 3), which may indicate item redundancy and data distribution problems. However, the item was retained because the model presented adequate indicators.

It was observed that all indices were within the reference values, indicating adequate quality of fit between the model and the sample data (Table 4).

The psychometric parameters of the model were satisfactory in terms of accuracy (ORION = 0.962), construct representativeness (FDI = 969), sensitivity (SR = 5.003), expected percentage (95.9%), and replicability by the latent G-H index (0.962) (Table 4).

The factor showed adequate reliability values, with a standardized Cronbach’s alpha of 0.78 and a McDonald’s omega of 0.75. The composite reliability was 0.81. Thus, all the indicators evaluated indicated a unidimensional model, with evidence of internal structure.

The internal consistency values observed (Cronbach’s alpha = 0.78; McDonald’s omega = 0.75; composite reliability = 0.81) indicate acceptable to good reliability, according to commonly adopted thresholds in psychometric research (≥0.70 for acceptable and ≥0.80 for strong reliability). These findings suggest that the instrument demonstrates satisfactory internal coherence, although slight variability among items may be present.

The Cronbach’s alpha value (0.78) was interpreted based on established benchmarks, in which values between 0.70 and 0.79 are considered acceptable, supporting the internal consistency of the instrument in this sample.

Table 5 shows the average responses for each item in the test and retest as well as the questionnaire’s reliability. In the test, the average ranged from 1.21 to 3.39, and in the retest, from 1.11 to 3.40. The weighted Kappa value ranged from 0.56 to 0.89. Most Kappa values showed a very good level of agreement, indicating a good understanding of the instrument’s questions.

The weighted Kappa coefficients indicated predominantly good to very good stability over time. However, items with lower Kappa values suggest potential variability in specific behaviors or measurement sensitivity, which should be interpreted cautiously and may reflect the dynamic nature of self-care practices.

## 4. Discussion

The DFSQ-UMA was developed and validated in the Spanish context, with the aim of assessing foot self-care in people with diabetes without amputations. Its use guides nurses’ decision-making in health education, monitoring, and health promotion initiatives for these patients [11]. Since its creation, the tool has been adapted to different cultures [15]. The present study adapted the instrument to the Brazilian context, demonstrating adequate psychometric properties and good reliability, indicating its potential applicability in the clinical practice of professionals in Brazil.

The results of the factor analysis indicated that the DFSQ-UMA presented a unidimensional/bifactorial structure, with adequate adjustment indices demonstrating theoretical consistency with the original model developed in Spain [11]. The observed unidimensionality confirms that the instrument consistently measures the construct “foot self-care,” encompassing self-care, self-management, self-examination, and footwear (shoes and socks).

Although the unidimensional structure supports the coherence of the construct, this finding also raises important theoretical and methodological considerations. The convergence of multiple domains into a single latent dimension may suggest conceptual overlap between items, indicating potential redundancy in the measurement of foot self-care behaviors. From a psychometric perspective, this result favors parsimony and simplifies score interpretation; however, it may also reduce the instrument’s sensitivity to distinguish between specific dimensions of self-care. Therefore, the retention of a unidimensional solution was based on empirical fit indices and theoretical interpretability, balancing statistical adequacy and conceptual clarity.

The findings of the present study are consistent with international adaptations of the DFSQ-UMA; however, some nuances deserve further consideration. While studies conducted in French [12], Italian [13], Persian [14], Arabic [15], English [16], and Turkish [17] contexts also reported adequate psychometric properties, differences in factor structure and item behavior suggest that cultural, social, and healthcare-related factors may influence how foot self-care is conceptualized and practiced. In this sense, the predominance of a unidimensional structure in the present study reinforces the generalizability of the construct, but also highlights the need to interpret results within specific contextual realities.

Transcultural adaptation is necessary to validate foot assessment tools for people with diabetes and ensure their use in different populations. This process involves not only translation, but also the modification of elements to represent local health practices and realities. The integration of these technologies into care enables the early identification of complications, thereby reducing unfavorable outcomes such as infections, amputations, and mortality [36].

When comparing the current version with international adaptations, a pattern of psychometric and cultural equivalence emerges. All versions demonstrated construct validity, good internal consistency, and high participant acceptability. The stability of the unidimensional structure of the DFSQ-UMA indicates that it is a widely accepted and applied construct in cross-cultural studies, with fundamental practices that remain consistent across cultures, although some behaviors may vary according to each country’s social and economic factors.

The methodological decision to retain the factorial structure was guided by multiple complementary criteria, including parallel analysis, goodness-of-fit indices, and indicators of unidimensionality. Although alternative factorial solutions were explored, the selected model demonstrated superior statistical performance and interpretability. Additionally, the use of both exploratory and confirmatory approaches within the same sample, while a recognized limitation, allowed a comprehensive evaluation of the internal structure in an initial validation context.

For this reason, the French study highlighted the importance of education in adherence to self-care practices [12], while the Italian study emphasized the role of family support and health education [13]. The Persian and Arabic versions, on the other hand, highlighted religiosity and socioeconomic conditions as determinants of self-care behavior [14,15].

These variations across studies reinforce that, although the core construct remains stable, contextual determinants such as health system organization, cultural beliefs, and socioeconomic conditions influence specific self-care behaviors. This highlights the importance of not only validating instruments but also interpreting their results within the sociocultural context in which they are applied.

By adapting an instrument to different languages and cultures, it is possible to investigate its results across contexts, enabling the development of common intervention, assessment, and monitoring strategies. The DFSQ-UMA has the potential to evaluate and monitor self-care practices, which are a key aspect of foot care. This evaluation enables the identification of educational needs, thereby contributing to the development and implementation of early and targeted strategies [16].

From a theoretical perspective, these findings contribute to the understanding of foot self-care as a potentially integrated construct rather than a strictly multidimensional one. Practically, this supports the use of a global score in clinical settings, facilitating decision-making and screening processes. However, clinicians should remain attentive to specific behaviors that may require targeted interventions, even when assessed within a unidimensional framework.

Foot ulceration in people with diabetes considerably reduces their health-related quality of life, impairing their daily activities. The assessment of specific dimensions related to foot health based on comprehensive and validated measures is necessary in care, to identify risk factors early and implement preventive strategies. The validation process, as evidenced by strong psychometric measures, ensures the application of the instrument in the clinical practice of health professionals when providing care to this population [37].

Adherence to foot self-care is a determining factor in the development of ulcers in patients with diabetes. Mekonen & Gebeyehu Demssie [38] identified that characteristics such as male gender, low education level, living in rural areas, presence of diabetes-related complications, use of injectable or tablet medications, lack of prior information about foot self-care, and little family support increase the chances of insufficient self-care. In these cases, health education practices, especially regarding regular foot inspection and appropriate footwear, are recommended during follow-up consultations.

The validation of the Brazilian version of the DFSQ-UMA-Br represents a significant advancement in the assessment of foot self-care in people with diabetes in the national context. The instrument can assist healthcare professionals, especially nurses and healthcare professionals, in the early identification of self-care deficits, allowing for the planning of more targeted educational and preventive interventions. In addition, the application of the DFSQ-UMA_Br in healthcare services can contribute to reducing complications, such as ulcers and amputations, and improving patients’ quality of life.

From a scientific perspective, the instrument enables cross-cultural comparisons across populations, expanding the body of evidence on self-care behavior and the effectiveness of educational strategies in diabetes. Thus, education should be structured, organized, and continuous, playing an important role in preventing diabetes-related foot ulcers [39].

Given this, the DFSQ-UMA-Br instrument enables healthcare professionals to recognize early self-care deficits in the feet and to direct effective educational interventions tailored to the patient’s needs, especially for those who need reinforced guidance and can benefit from systematic health education.

Despite the strengths of this study, some limitations should be critically acknowledged. The use of convenience sampling may introduce selection bias, although data were collected across three specialized centers to increase variability. Furthermore, the absence of similar validated instruments in Brazil limited the assessment of convergent and discriminant validity. Finally, the cross-sectional design restricts the ability to assess temporal stability beyond the test–retest approach.

Some methodological aspects should be considered when interpreting these findings. The use of a non-probabilistic sampling strategy may limit the generalizability of the results, although efforts were made to include participants from multiple specialized centers. Additionally, the cross-sectional design does not allow causal inferences or evaluation of changes over time in self-care behaviors. These factors should be taken into account when extrapolating the results to other populations.

## 5. Conclusions

The Brazilian version of the Diabetic Foot Self-Care Questionnaire—University of Malaga (DFSQ-UMA-Br) demonstrates adequate psychometric properties, with evidence of construct validity and reliability, making it a valid and reliable instrument for assessing foot self-care in people with diabetes in Brazil. Its use may strengthen evidence-based clinical practices and support the monitoring and improvement of health education actions aimed at preventing foot complications in individuals with diabetes. It can be used by nurses and other healthcare professionals to promote health in both clinical practice and research.

## Figures and Tables

**Table 1:** Distribution of descriptive data (n = 269).

Items	Mean	95% CI	Variance	Asymmetry (sk)	kurtosis (ku)
1	2.746	2.52–2.97	2.050	0.467	−1.152
2	2.279	2.03–2.53	2.613	0.778	−1.092
3	2.088	1.85–2.33	2.382	1.104	−0.446
4	1.864	1.69–2.04	1.213	0.934	−0.226
5	2.768	2.54–2.99	2.112	0.206	−1.145
6	1.813	1.58–2.05	2.270	1.461	0.300
7	2.724	2.52–2.93	1.788	0.292	−1.653
8	2.000	1.82–2.18	1.382	0.667	−1.046
9	1.951	1.79–2.11	1.090	1.147	0.955
10	2.132	1.12–1.30	0.344	2.877	2.637
11	2.820	2.52–3.12	3.633	0.199	−1.885
12	2.103	1.90–2.30	1.636	0.906	−0.299
13	3.393	3.13–3.65	2.827	0.462	−1.490
14	1.217	1.10–1.33	0.530	3.655	3.068
15	2.669	2.54–2.79	0.641	0.973	1.131
16	1.349	1.18–1.52	1.154	2.914	3.764

95% CI: 95 percent confidence interval.

**Table 2:** Dimensionality analysis (n = 269).

Items	I-UniCo	95% CI	I-ECV	95% CI	MIREAL	95% CI
1	0.999	0.980–1.000	0.963	0.830–0.998	0.177	0.043–0.373
2	1.000	0.994–1.000	0.985	0.898–1.000	0.116	0.008–0.299
3	0.998	0.982–1.000	0.941	0.839–0.994	0.227	0.070–0.379
4	1.000	1.000–1.000	1.000	1.000–1.000	0.001	0.000–0.001
5	0.999	0.963–1.000	0.959	0.780–1.000	0.118	0.003–0.265
6	1.000	1.000–1.000	0.999	0.995–1.000	0.012	0.000–0.028
7	0.991	0.742–1.000	0.881	0.725–0.998	0.168	0.011–0.381
8	0.977	0.900–1.000	0.889	0.800–0.979	0.128	0.088–0.356
9	0.952	0.930–1.000	0.856	0.259–0.999	0.158	0.088–0.384
10	0.999	0.939–1.000	0.950	0.854–1.000	0.051	0.000–0.188
11	0.942	0.900–1.000	0.840	0.800–0.358	0.845	0.478–0.998
12	0.998	0.912–1.000	0.883	0.811–0.803	0.348	0.114–0.626
13	0.956	0.900–1.000	0.853	0.800–0.266	0.807	0.647–0.979
14	1.000	0.994–1.000	0.990	0.902–1.000	0.032	0.001–0.103
15	1.000	1.000–1.000	0.999	0.997–1.000	0.006	0.001–0.009
16	1.000	1.000–1.000	0.999	0.999–1.000	0.003	0.000–0.001
UniCo	0.994	0.734–0.999	-	-	-	-
ECV	-	-	0.857	0.783–0.992	-	-
MIREAL	-	-	-	-	0.200	0.183–0.206

Source: Prepared by the authors.

**Table 3:** Factor structure of the DFSQ-UMA-Br model (n = 269).

Items		F1	h^2^
1	Do you usually evaluate your feet?	0.846	0.870
2	Do you check for wounds or examine the skin on your feet?	0.849	0.884
3	Do you check your nails? Don’t answer if all your toes have been amputated.	0.761	0.898
4	How important is it to you to take care of your feet regularly?	0.753	0.591
5	What about guidelines on how you should take care of your feet?	0.532	0.442
6	What do you do when your feet are dry and calloused?	0.444	0.502
7	To dry your feet:	0.399	0.834
8	Is it difficult to find comfortable shoes for your feet?	0.920	0.821
9	How often do you trim or care for your toenails? Don’t answer if you have had all your toes amputated.	0.806	0.791
10	Is it difficult for you to dry your feet after showering? Don’t answer if you have had all your toes amputated.	0.485	0.752
11	Do you have trouble finding socks that fit your feet?	0.864	0.740
12	Regarding the shoes you wear every day, before putting them on:	0.364	0.657
13	Regarding socks:	0.837	0.698
14	Regarding new shoes:	0.402	0.893
15	On very hot days, what type of shoes do you wear?	0.398	0.636
16	To warm your feet:	0.632	0.605

F: Factor; h^2^: commonalities.

**Table 4:** Model fit quality; quality and effectiveness of estimates (n = 269).

Indexes	Values Obtained	Reference Values
Tucker–Lewis Index (TLI)	0.966 (0.965–0.977)	>0.90
Comparative Fit Index (CFI)	0.975 (0.974–0.983)	>0.94
Goodness of Fit Index (GFI)	0.955 (0.948–0.969)	>0.95
Standardized root mean square residuals (SRMRs)	0.069 (0.061–0.072)	<0.07
Root Mean Square Error of Approximation (RMSEA)	0.043 (0.032–0.046)	<0.07
Overall Reliability of Fully Informative Prior Oblique AND AP Scores (ORION)	0.962	>0.90
Factor Determinacy Index (FDI)	0.969	>0.80
Sensitivity Ratio (SR)	5.003	>2.0
Expected Percentage of true Differences (EPTD)	95.9%	>90%
Generalized G-H Index	0.962	>0.80

Source: Prepared by the authors.

**Table 5:** Reliability of the DFSQ-UMA-Br items.

Items	Mean Test (SD)	Mean Retest (SD)	Weighted Kappa	95% CI	*p*-Value
01	2.75 (1.43)	2.63 (1.02)	0.66	0.53–0.72	0.65
02	2.28 (1.62)	2.01 (1.55)	0.88	0.78–0.92	0.61
03	2.09 (1.55)	2.05 (1.00)	0.64	0.59–0.68	0.77
04	1.86 (1.10)	1.87 (1.05)	0.78	0.72–0.84	0.62
05	2.77 (1.46)	2.78 (1.34)	0.69	0.62–0.73	0.88
06	1.81 (1.51)	1.68 (1.01)	0.56	0.54–0.61	0.89
07	2.72 (1.34)	2.69 (1.38)	0.78	0.71–0.83	0.67
08	2.00 (1.18)	1.88 (0.89)	0.71	0.62–0.77	0.78
09	1.95 (1.05)	1.76 (1.02)	0.69	0.62–0.74	0.89
10	1.21 (0.59)	1.11 (0.28)	0.82	0.78–0.88	0.67
11	2.82 (1.91)	2.79 (1.86)	0.71	0.62–0.77	0.90
12	2.10 (1.28)	2.05 (1.20)	0.62	0.56–0.66	0.55
13	3.39 (1.68)	3.40 (1.57)	0.74	0.69–0.76	0.58
14	1.22 (0.73)	1.14 (0.69)	0.89	0.83–0.91	0.62
15	2.67 (0.80)	2.70 (0.76)	0.84	0.78–0.89	0.76
16	1.35 (1.08)	1.36 (1.03)	0.69	0.62–0.74	0.70

Source: Prepared by the authors.

## Data Availability

The data presented in this study are available on reasonable request from the corresponding author. The data are not publicly available due to privacy and ethical restrictions.

## Public Involvement Statement

Participants from the target population were involved in this research by taking part in the validation process of the instrument. Their responses contributed to the assessment of the psychometric properties of the Brazilian version of the Diabetic Foot Self-Care Questionnaire (DFSQ-UMA-Br). No members of the public were involved in the design, conduct, reporting, or dissemination plans of this research.

## Guidelines and Standards Statement

This manuscript was drafted in accordance with recommended standards for studies on psychometric evaluation and cross-cultural adaptation of measurement instruments.

## Abbreviations

The following abbreviations are used in this manuscript:

DFSQ-UMA	Diabetic Foot Self-Care Questionnaire of the University of Malaga-Spain
DFSQ-UMA-Br	Brazilian version of the Diabetic Foot Self-Care Questionnaire of the University of Malaga-Spain
DFSQ-UMA-En	English version of the Diabetic Foot Self-Care Questionnaire of the University of Malaga-Spain
DFSQ-UMA-FR	French version of the Diabetic Foot Self-Care Questionnaire of the University of Malaga-Spain
DFSQ-AR	Arabic version of the Diabetic Foot Self-Care Questionnaire
T2DM	Type 2 Diabetes Mellitus
UECE	State University of Ceará
